# Supplementary material for: Single Cell Transcriptomic and Chromatin Profiles Suggest Layer Vb Is the Only Layer With Shared Excitatory Cell Types in the Medial and Lateral Entorhinal Cortex
Source: Front Neural Circuits. 2022 Jan 26;15:806154. doi: 10.3389/fncir.2021.806154 (PMC8826650; doi:10.3389/fncir.2021.806154)
Supplement: Supplementary file 14 [file Data_Sheet_1.ZIP › Exc_vs_Inh_Motifs/homerResults/motif34.similar.html]

motif34

## Information for motif34

A
G
T
C
A
C
T
G
A
C
G
T
A
C
G
T
A
C
T
G
A
C
T
G
A
C
G
T
A
G
T
C
  
Reverse Opposite:  

A
C
T
G
C
G
T
A
A
G
T
C
A
G
T
C
C
G
T
A
C
G
T
A
A
G
T
C
A
C
T
G
  

|  |  |
| --- | --- |
| p-value: | 1e-24 |
| log p-value: | -5.654e+01 |
| Information Content per bp: | 1.530 |
| Number of Target Sequences with motif | 89.0 |
| Percentage of Target Sequences with motif | 0.21% |
| Number of Background Sequences with motif | 6.9 |
| Percentage of Background Sequences with motif | 0.06% |
| Average Position of motif in Targets | 310.0 +/- 211.2bp |
| Average Position of motif in Background | 347.4 +/- 184.0bp |
| Strand Bias (log2 ratio + to - strand density) | -0.1 |
| Multiplicity (# of sites on avg that occur together) | 1.00 |
| Motif File: | file (matrix) reverse opposite |

### Similar de novo motifs found

|  |  |  |  |  |  |  |  |
| --- | --- | --- | --- | --- | --- | --- | --- |
| Rank | Match Score | Redundant Motif | P-value | log P-value | % of Targets | % of Background | Motif file |
